# The role of a lens survival pathway including *sox2* and *αA-crystallin* in the evolution of cavefish eye degeneration

**DOI:** 10.1186/2041-9139-5-28

**Published:** 2014-08-28

**Authors:** Li Ma, Amy Parkhurst, William R Jeffery

**Affiliations:** 1 Department of Biology, University of Maryland, College Park, MD 20742, USA

**Keywords:** *Astyanax mexicanus*, Blind cavefish, Lens apoptosis, Eye degeneration, *αA-crystallin*, *Sox2*, Cis and trans gene regulation, Lens survival pathway

## Abstract

**Background:**

The teleost *Astyanax mexicanus* is a single species consisting of eyed surface-dwelling (surface fish) and blind cave-dwelling (cavefish) morphs. Cavefish eyes are lost through apoptosis of the lens, which in turn promotes the degeneration of other optic tissues. The *αA-crystallin* (*αA-crys*) gene is strongly downregulated in the cavefish lens and is located in a genomic region (QTL) responsible for eye loss. Therefore, *αA-crys* has been proposed as a candidate for regulating cavefish eye degeneration. The purpose of this study was to determine the mechanism of *αA-crys* downregulation and its role in cavefish eye degeneration.

**Results:**

The involvement of *αA-crys* in eye degeneration was confirmed by knocking down its expression in surface fish, which led to apoptosis of the lens. The underlying reason for *αA-crys* downregulation in cavefish was investigated by comparing genomic *αA-crys* DNA sequences in surface fish and cavefish, however, no obvious cis-regulatory factors were discovered. Furthermore, the cavefish *αA-crys* allele is expressed in surface fish x cavefish F1 hybrids, indicating that evolutionary changes in upstream genes are most likely responsible for *αA-crys* downregulation. In other species, Sox2 is one of the transcription factors that regulate lens *crystallin* genes during eye development. Determination of *sox2* expression patterns during surface fish and cavefish development showed that *sox2* is specifically downregulated in the cavefish lens. The upstream regulatory function of Sox2 was demonstrated by knockdown in surface fish, which abolished *αA-crys* expression and induced lens apoptosis.

**Conclusions:**

The results suggest that *αA-crys* is required for normal eye development in cavefish via suppression of lens apoptosis. The regulatory changes involved in *αA-crys* downregulation in cavefish are in trans-acting factors rather than cis-acting mutations in the *αA-crys* gene. Therefore, *αA-crys* is unlikely to be the mutated gene(s) associated with an *Astyanax* eye QTL. The results reveal a genetic pathway leading from *sox2* to *αA-crys* that is required for survival of the lens in *Astyanax* surface fish. Defects in this pathway may be involved in lens apoptosis and thus a cause of cavefish eye degeneration.

## Background

The loss of eyes is one of the hallmarks of cave-adapted animals [1]. The mechanisms of eye loss have been studied in the teleost *Astyanax mexicanus*, which has an ancestral eyed surface-dwelling morph (surface fish) and multiple derived cave-dwelling morphs (cavefish) with reduced or absent eyes [2-5]. The surface fish and cavefish morphs spawn frequently in the laboratory and are interfertile, allowing developmental and genetic analysis to be used to explore the evolutionary mechanisms of eye degeneration. Eye loss in cavefish is a developmental process [6]. Both *Astyanax* morphs develop eye primordia during embryogenesis. After hatching the eye primordia continue to develop and grow in surface fish but growth is arrested in cavefish, and the eyes degenerate and sink into the orbit.

Apoptosis of the lens is considered to be a primary cause of cavefish eye degeneration [7-10]. Accordingly, eye development and growth can be rescued by replacing the apoptotic cavefish lens with a surface fish embryonic lens [8], showing that the lens controls the development of the surrounding eye tissues. Eye loss in cavefish is a multigenic trait, and several significant quantitative trait loci (QTL) have been discovered that are responsible for the degenerative eye phenotype, including arrested development of the lens [11-15]. Mapping of *Astyanax* QTL to the zebrafish genome [13], and more recently the cavefish genome [McGaugh and 20 others 2014, The cavefish genome reveals candidate genes for eye loss, In submission], has shown that the *αA-crystallin* (*αA-crys*) locus is linked to an eye QTL. Furthermore, *αA-crys* expression is strongly downregulated in cavefish [16,17].

The molecular chaperone α-crystallin is a member of the small heat-shock protein family. It consists of the αA- and αB-crystallin (*αB-crys*) subunits, which function as anti-apoptotic proteins [18-20]. α-crystallin prevents apoptosis by inhibiting procaspase-3 activation and binding to Bax so that it cannot translocate to mitochondria [21]. In a number of vertebrate species, the *αA-crys* gene is specifically expressed in the lens beginning at the time of lens fiber cell elongation [22,23]. Analysis of the zebrafish *cloche* mutant shows that *αA-crys* is required for normal lens development. In the absence of *αA-crys*, *γ-crystallin* is not solubilized and lens fiber cells fail to differentiate, which affects lens transparency and can produce cataracts [24]. As a survival protein, α-crystallin prevents the completion of an apoptosis-like program that is normally initiated in the lens fiber cells to eliminate their organelles [25,26]. In the *αA*/*αB*-crystallin double knockout mouse, the lens is significantly smaller than in the wild type and fiber cell formation is severely disrupted [27].

Lens-specific expression of the *αA-crys* gene is regulated primarily at the transcriptional level [28]. *αA-crys* promoter regions and cis-acting enhancers that bind transcription factors, such as Pax6, CREB, and USF, have been identified in the mouse and chicken [28]. The *Astyanax αA-crys* gene has also been cloned and sequenced [16]. Despite its strong downregulation in cavefish, only minor changes in the coding region, an intron, and a part of the 5’ non-coding region including the putative promoter were detected between surface fish and cavefish [16]. However, only a relatively small region of the 5’ region flanking the *αA-crys* promoter was sequenced [16], and therefore any differences in sequences reflecting cis-acting regulatory changes located further upstream or in the 3’ non-coding region would not have been detected. Thus the molecular basis for *αA-crys* downregulation in the cavefish lens is currently unknown.

During lens development, the *crystallin* genes are regulated by a complex array of transcription factors, including Pax6, retinoic acid receptors, members of the Sox, Maf, and CREB families, AP-1, and Prox1 [29,30]. Pax6 binds directly to enhancer sequences and activates expression of the chicken *αA-crys* and *δ- crystallin*, mouse *αA-crys* and *αB-crys*, and guinea pig *ξ crystallin* genes [28]. In mice, tissue-specific *αA-crys* expression in the lens is regulated via the recruitment of Pax6 and c-Maf to its promoter [31]. Pax6 also has a role in cavefish eye degeneration [7]. At the early neurula stage, the *pax6* expression domains corresponding to the eye primordia are smaller in cavefish embryos, and at later stages surface fish larvae have stronger *pax6* expression in the eye than cavefish larvae [32]. The *pax6* expressing eye domains appear to be negatively regulated by overexpression of *shh* and related genes along the cavefish embryonic midline [33].

The *sox2* gene is also important in lens and eye development. In humans and mice, *sox2* is widely expressed during brain and spinal cord development [34]. In addition, *sox2* expression is observed in the developing eye, particularly in the lens, neural retina, and optic nerve [35-37]. Sox2 generally exhibits gene regulatory functions by forming complexes with partner transcription factors, and the binding of a single Sox protein alone to a DNA site does not lead to transcriptional activation or repression [38,39]. In many species, Pax6 and Sox2 regulate *crystallin* gene expression cooperatively [40,41]. For example, Sox2 has been shown to bind cooperatively with Pax6 to the *δ-crystallin* minimal enhancer DC5 [42]. When transfected into chick embryos, *sox2* alone is not sufficient to induce ectopic lens tissue; however, when co-mis-expressed with *pax6*, lens tissue is induced cell-autonomously in surface ectoderm outside of the eye [42]. Despite its importance in vertebrate lens and eye development, the *sox2* gene has not been previously investigated in *Astyanax mexicanus*.

In this investigation, we have explored the role of *αA-crys* and *sox2* genes in lens apoptosis and eye degeneration through gene expression, gene knockdown, and genetic analysis. Our results reveal that *sox2* functions upstream of *αA-crys* in a genetic pathway that is required for lens survival in surface fish. The disruption of the *sox2*-*aA-crys* pathway may be one of the causes of lens apoptosis and eye degeneration in cavefish.

## Methods

### Biological materials

These experiments used two populations of *Astyanax mexicanus* surface fish and six populations of cavefish. The surface fish populations were raised in the laboratory from fish originally collected at Balmorhea State Park, Texas (Texas surface fish) and Nacimiento Del Rio Choy, San Luis Potosi, Mexico (Mexican surface fish). The cavefish populations included four laboratory-raised strains originally collected at Cueva de El Pachón in Tamaulipas, Mexico (Pachón cavefish), El Sotano de la Tinaja (Tinaja cavefish) and Cueva de los Sabinos (Los Sabinos cavefish), both in San Luis Potosi, Mexico, and La Cueva Chica (Chica cavefish) originally provided by the Steinhardt Aquarium (San Francisco, CA USA), and two populations collected from El Sotano de Molino (Molino cavefish) and El Sotano de Jineo (Jineo cavefish) in Tamaulipas, Mexico. Unless specifically named in the text, experiments referring to surface fish imply the Texas population, and those referring to cavefish imply the Pachón population. The fish collections were done under the auspices of Mexican Permit Number 040396-213-03.

### Fish maintenance and embryo collection

Fish were maintained in the laboratory at 22.5°C on a 14-hr light and 10-hr dark photoperiod [7,43]. Natural spawning or *in vitro* fertilization was used to obtain Texas surface fish and Pachón cavefish embryos. F1 hybrid embryos were obtained by *in vitro* fertilization in both directions. Some embryos were cultured with 400 μM phenylthiourea (PTU) to remove pigmentation prior to *in situ* hybridization (see below) as described previously [44].

### RNA isolation, cDNA synthesis, and RT-PCR

Total embryonic RNA was isolated with TRI Reagent Solution (Life Technologies, Grand Island NY, USA), and cDNA was synthesized using the SuperScriptTM III First-Strand Synthesis SuperMix Kit and oligo (dT)_20_ primers (Life Technologies). Semiquantitative RT-PCR was done using the PCR Master kit (Roche, Germany). The primers for amplification of *aA-crys* by semiquantitative RT-PCR were 5’-TTTGACTATGACCTCTTCCCCTACGC-3’ (forward) and 5’-GGGGGTAGAGTTAGTCTTGTCGTCAC-3’ (reverse). The PCR cycling conditions were one cycle of initial denaturation at 94°C for 2 minutes, followed by 32 cycles each of denaturation (94°C for 30 sec), annealing (at 64°C for 30 sec), and elongation (at 72°C for 30 sec) with a final elongation step at 72°C for 7 minutes. The primers for amplification of *sox2* by semiquantitative RT-PCR were 5’-CTGCACATGAAGGAACACCC-3’ (forward) and 5’-GACATGCTGTAGGTGGGCGA-3’ (reverse), and the PCR cycling conditions were one cycle of initial denaturation at 94°C for 2 minutes, 25 cycles of denaturation (94°C for 30 sec), annealing (at 60°C for 30 sec), and elongation (at 72°C for 30 sec), followed by a final elongation step at 72°C for 7 minutes. For semiquantitative RT-PCR, 18S rRNA was used as the standard. The primers for 18S rRNA were 5’-GAGTATGGTTGCAAAGCTGAAA-3’ (forward) and 5’-CCGGACATCTAAGGGCATCA-3’ (reverse).

### Genome walking and sequencing

Using the *Astyanax αA-crys* genomic sequence (GenBank Y11301.1), a 10 kb *αA-crys* genomic DNA sequence was amplified from surface fish and Pachón cavefish using the GenomeWalker™ universal kit (Clontech Labotatories, Mountain View, CA, USA). For the construction of GenomeWalker libraries, surface fish and Pachón cavefish genomic DNA was digested with *EcoR* V, *Dra* I, *Pvu* II, and *StuI* I. The GenomeWalker reactions were performed with TaKaRa LA Taq™ (TAKARA Bio INC). The 5’sequence upstream of the *αA-crys* coding region was amplified five times, step by step, with the following primer sequences: in the first amplification, the primary PCR primer was 5’-TTGCGGAAGAGCGAGTACCGATAATAA-3’, and the nested PCR primer was 5’-CGTAGGGGAAGAGGTCATAGTCAAACA-3’; in the second amplification, the primary primer was 5’-GTGCACACTGGTACACACTGTCATTTAG-3’, and the nested primer was 5’-GTGCCCAACTACTTTAGTTCTGATTGTC-3’; in the third amplification, the primary primer was 5’-CTTGGCAATATCTGGAATTCAGTAGAC-3’, and the nested primer was 5’-CCCAATTAGCTCAATAACATCCTTGAC-3’; in the fourth amplification, the primary PCR primer was 5’-CTGCCACCCTGATCCTGCATCCGATGA-3’, and the nested primer was 5’-GGGTCTAGGTCAGGCCATTCATTATC-3’; and finally in the fifth amplification, the primary PCR primer was 5’-GGTCTGTTAACTGCTGTGTGTCCTTGT-3’, and the nested primer was 5’-TTCTAATCAGTCAGTAGTGCACCTGTG-3’. The PCR primers used for amplification of the 3’sequence downstream of the *αA-crys* coding region were 5’-CTCCAACGTGGACCAGTCGGCCATCA-3’ (primary), and 5’-TCCTGTCACCCGTGACGACAAGACTAA-3’ (nested). The PCR reactions were conducted using the two-step cycle parameters described in the GenomeWalker™ universal kit manual (Clontech). After obtaining the major bands, the fragments were cloned into the TOPO TA clone vector (Life Technologies), sequenced using M13 or M13-20 primers, and the results were deposited in Genbank [KJ786414, KJ786415].

### RACE amplification

The SMARTer™ RACE cDNA Amplification kit (Clontech) was used for 5’ RACE and 3’RACE amplification of the surface fish and Pachón cavefish *sox2* genes. Poly A^+^ RNA from surface fish and Pachón cavefish was isolated with the NucleoBond RNA/DNA kit (Macherey-Nagel, Duren, Germany) and RACE-Ready cDNA was generated using the first-strand cDNA synthesis protocol in the SMARTer™ RACE cDNA Amplification kit (Clontech). The primary gene-specific primer for *Astyanax sox2* 5’RACE was 5’-TGGCGGCGGGGTGGGCGTCGGTGTT-3’; the nested gene-specific primer for *sox2* 5’RACE was 5’-TTGGCTCCGGCGTTGGGGCCGGCAT-3’; the primary gene-specific primer for *Astyanax sox2* 3’RACE was 5’-CGTCTGCGCGCTGGTCATGGAGCCGTA-3’; and the nested gene-specific primer for *sox2* 3’RACE was 5’-GGGCGCGTTGAGGCCGGCGTGCTGC-3’. The PCR reactions were performed with the Advantage 2 PCR Kit (Clontech Laboratories). The PCR conditions were five cycles of 94°C for 30 sec and 72°C for 3 minutes; 5 cycles of 94°C for 30 sec, 70°C for 30 sec, and 72°C for 3 minutes, 27 cycles of 94°C for 30 sec, 68°C for 30 sec, and 72°C for 3 minutes. For the nested PCR reactions, the cycling conditions were 20 cycles each of 94°C for 30 sec, 68°C for 30 sec, and 72°C for 3 minutes. The PCR products were purified with the MinElute PCR Purification Kit (Qiagen, Valencia, CA, USA) and sequenced with nested gene-specific primers. The surface fish and Pachón cavefish *sox2* sequences were deposited in Genbank [KJ812146, KJ812147, respectively].

### PCR amplification of α*A-crys* DNA regions with sequence differences in multiple surface fish and cavefish populations

**Figure 1:**
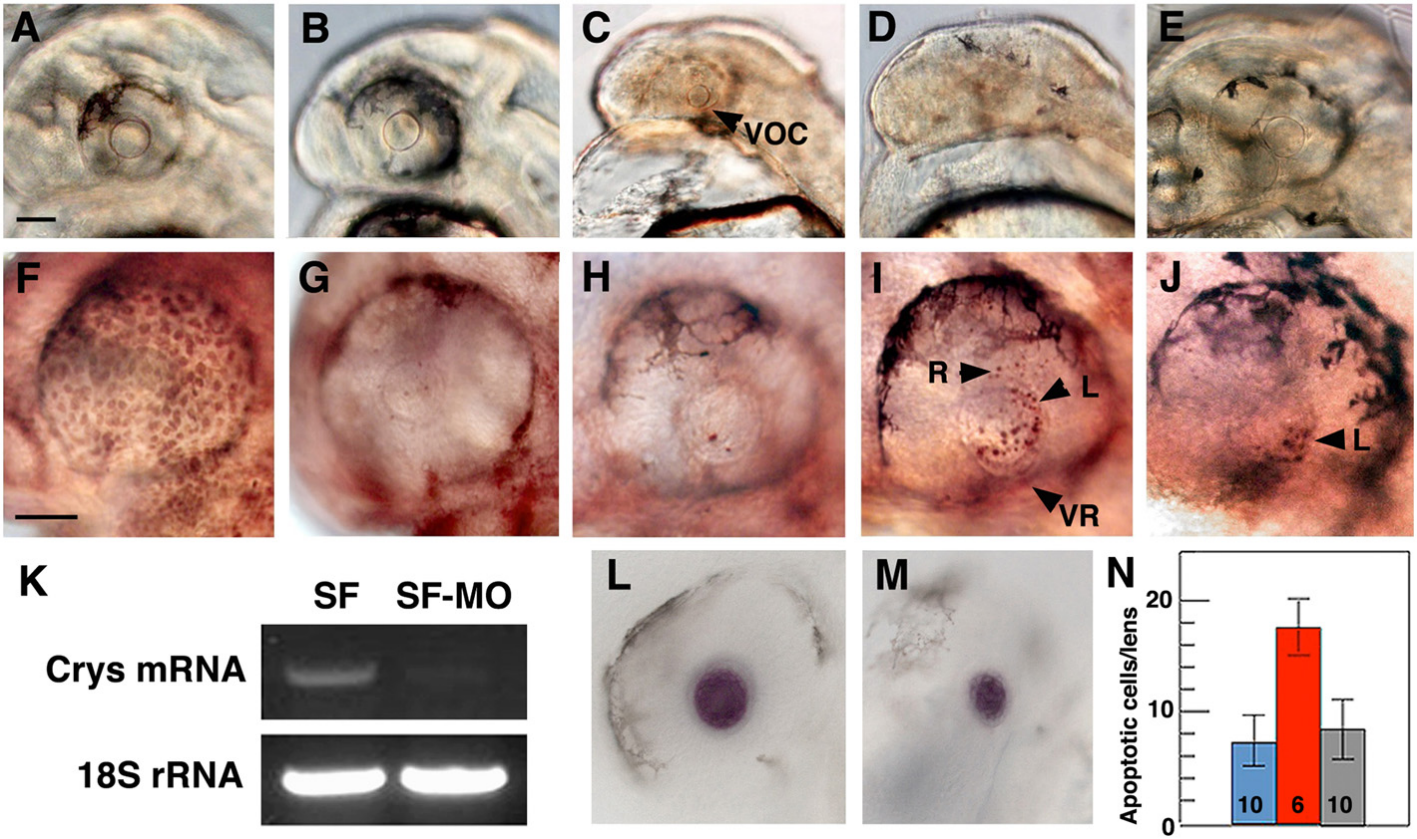
***αA-crys *knockdown with morpholinos (MOs). (A-E)** Effects of MO on lens development. **(A)** Embryos injected with control MO have a normal sized lens. **(B-D)** Embryos injected with *αA-crys* splice-blocking morpholinos (sbMO) and translation-blocking morpholino (tbMO) develop a normal sized lens **(B)**, a lens of reduced size and a reduced ventral optic cup (VOC) **(C)1**, or no lens **(D)**. **(E)** Embryos injected with *αA-crys* sbMO and *αA-crys* mRNA show normal lens development. **(F-J)** Apoptosis detected by terminal deoxynucleotidyl transferase dUTP nick end labeling (TUNEL). **(F)** An uninjected embryo treated with DNase shows apoptotic cells throughout the eye. **(G, H)** Uninjected **(G)** and control MO-injected **(H)** embryos show background levels of lens apoptosis. **(I)** Embryos injected with *αA-crys* sbMO and tbMO embryos show apoptotic cells in the lens (L) and retina (R). VR: reduced ventral retina. **(J)** Embryos injected with *αA-crys* sbMO and *αA-crys* mRNA show background levels of lens apoptosis. **(K)** Semiquantitative RT-PCR . SF: uninjected embryos. SF-MO: *αA-crys* MO-injected embryos. **(L, M)***In situ* hybridization shows *mip* gene expression in the lens of uninjected **(L)** and *αA-crys* sbMO-injected **(M)** embryos. All embryos are shown at 40 hr post fertilization (hpf). All scale bars are 80 μm; **A-E, F-J**, **L** and **M** are the same magnifications. **(N)** Histogram showing lens apoptotic cells in control MO-injected embryos (blue), *αA-crys* sbMO-injected embryos (red), and *αA-crys* sbMO and *αA-crys* mRNA-injected embryos (gray). Error bars represent SD. Numbers at the base of the histogram represent sample sizes. *P* = 0.00 (one-way analysis of variance (ANOVA).

Total DNA was extracted from fin clips of multiple surface fish and cavefish populations using the DNeasy Blood & Tissue Kit (Qiagen). The four regions of DNA with major sequence differences (see Figure 1A) were amplified with the Phusion High-Fidelity PCR Master Mix (New England Biolabs, Ipswich, MA, USA). The primers used to amplify the sequence change 1 (SC1) region were 5’-GCCTGCATGTGCCAGAGGGG-3’ (forward) and 5’-CCGCCGCCAAAACATTGCGT-3’ (reverse). The PCR cycling conditions were one cycle of initial denaturation at 98°C for 30 sec, followed by 35 cycles of denaturation (98°C for 5 sec), annealing (at 60°C for 15 sec), extension (at 72°C for 15 sec), and a final extension at 72°C for 7 minutes. The primers used to amplify the sequence change 2 (SC2) region were 5’-TGGGAGGCCCTGATGCACAACT-3’ (forward) and 5’-TTGGATGTTTGTTGGGCTGTGTGC-3’ (reverse). The PCR cycling conditions were similar to those used to amplify SC1 except that annealing was performed at 66°C for 15 sec. The primers used to amplify the sequence change 3 (SC3) region were 5’-CCAGAGGCAGACATGTTTCCGATT-3’ (forward) and 5’-GGAGGCTGCAGAGTACTGACAGT-3’ (reverse). The PCR cycling conditions were the same as those used to amplify SC2. The primers used to amplify the sequence change 4 (SC4) region were 5’-TGGCTTCAAGCAAGGGCGGG-3’ (forward) and 5’-AGTTGCGGGCAACATCATACCCT-3’ (reverse). The PCR cycling conditions were similar to those used to amplify SC1 except that annealing was done at 58°C for 15 sec. The PCR products were purified with the MinElute PCR Purification Kit (Qiagen) and sequenced.

### PCR amplification of the *α*A-crys coding region from surface fish, Pachón cavefish, and F1 hybrid embryos for sequencing

The entire *aA-crys* coding region was PCR-amplified from surface fish, Pachón cavefish, and F1 hybrid embryos using the primers 5’-AGGCAGAGATTCGCCAAGAC-3’ (forward) and 5’-AAGTCGGGAGAGGGCTAAGT-3’ (reverse). The PCR reaction was performed using the Phusion High-Fidelity PCR Master Mix (New England BioLabs). The PCR cycling conditions were 1 cycle of initial denaturation at 98°C for 30 sec, followed by 35 cycles each of denaturation (98°C for 5 sec), annealing (at 60°C for 20 sec), and extension (at 72°C for 30 sec) with a final elongation step at 72°C for 10 minutes. The PCR products were purified with the MinElute Gel Extraction Kit (Qiagen) and sequenced using the forward PCR primer 5’-AGGCAGAGATTCGCCAAGAC-3’. The resulting 745-bp PCR product was cloned into the Dual Promoter PCR II-TOPO Vector in the TOPO TA Cloning Kit (Life Technologies) and sequenced using primers M13-20 and M13rev.

### Probe preparation and whole-mount *in situ* hybridization

The coding region fragments used as *αA-crys*, *sox2,* and *major lens intrinsic protein* (*mip*) probes were amplified from surface fish cDNA. For the *αA-crys* probe 5’-TTTGACTATGACCTCTTCCCCTACGC-3’ (forward) and 5’-GGGGGTAGAGTTAGTCTTGTCGTCAC-3’ (reverse) primers were used. Two coding region probes, *sox2*-A and *sox2*-B, were amplified and cloned: the primers for *sox2*-A were 5’-CTGCACATGAAGGAACACCC-3’ (forward) and 5’-GACATGCTGTAGGTGGGCGA-3’ (reverse), and for *sox2*-B were 5’-AGCCGTCCATTCTCTGGTTC-3’ (forward) and 5’-CTTGGTCGAGTGGAGAAGGTT-3’ (reverse). The primers for *mip* gene amplification (AF264702.1) were 5’-ACTTTTGCCTTCCTGATCGGT-3’ (forward) and 5’-AGGTGTCCCATGAGCACAGA-3’ (reverse). The resulting PCR products were cloned into the TOPO TA Dual Promoter cloning vector (Life Technologies) and confirmed by sequencing.

*In situ* hybridization was performed according to Bilandzija *et al*. [44] with modifications. Sense and anti-sense digoxygenin (DIG)-labeled RNA probes for *Astyanax αA-crys*, *sox2*, and *mip* were transcribed with SP6 and T7 RNA polymerase (Roche). The embryos were fixed with 4% paraformaldehyde (PFA) in PBS overnight at 4°C, dehydrated in an increasing methanol series, and stored at -20°C. Rehydrated embryos were treated with Proteinase K (10 μg/ml in PBS plus 0.1% Tween (PBST) for 5 to 10 minutes at room temperature, washed twice with PBST, post-fixed for 20 minutes with 4% PFA in PBST, and washed five times with PBST (5 minutes each). The embryos were pretreated with HYB- (50% formamide, 5 × SSC, 0.1% Tween-20) for 5 minutes. at 60°C. The HYB- was replaced with HYB + (HYB-, 1 mg/ml yeast RNA, 50 μg/ml heparin), and the embryos were pre-hybridized at 60°C for 4 hr. The pre-hybridization mix was removed and replaced with 1 ng/μl of sense or anti-sense probe in HYB+. Hybridization was carried out at 60°C overnight. The embryos were then washed twice at 60°C with 50% formamide, 2 × SSCT (saline sodium citrate plus 0.1% Tween-20) for 30 minutes each, once with 2 × SSCT (15 minutes) at 60°C), twice with 0.2 × SSCT (20 minutes each) at 60°C, and twice with MABT (150 mM maleic acid, 100 mM NaCl, pH7.5, 0.1% Tween-20) for 5 minutes each at room temperature. The embryos were incubated in blocking solution (MABT, 2% blocking reagent) overnight at 4°C and then with Anti-Digoxigenin-AP Fab fragments (1:5,000) (Roche) in blocking solution overnight at 4°C. The embryos were washed once with MABT containing 10% sheep serum at room temperature for 25 minutes, and eight more times (45 to 60 minutes each) with MABT at room temperature. Then, the embryos were washed with PBST and incubated in BM Purple AP Substrate (Roche) at room temperature in the dark. After the signal developed, the embryos were processed through an increasing glycerol series in PBS (30% to 50% to 80%) and imaged by microscopy.

For histology, *in situ* hybridized embryos were dehydrated through an ethanol series, cleared in Histo-Clear (National Diagnostics, Atlanta, GA, USA) and sectioned at 10 μm thickness. The sections were placed in glass microscope slides and stained with eosin.

### Gene knockdown procedures

To knock down the *αA-crys* gene, a cocktail containing 0.25 mM translation-blocking morpholino (tbMO: 5’-ATGGCAATATCCATAATGACTGGGC-3’) and 0.25 mM splice-blocking morpholino (sbMO: 5’AATGAGGTTCGAAGGCTTACCTGTC-3’) was injected into 1- to 4-cell-stage surface fish embryos. The sbMO was designed against *αA-crys* exon 2 [45]. To knockdown the single exon *sox2* gene, the tbMO 5’-GTCAGCAGAGCGGACCCCCCATGAC-3’ was used, which was designed according to the sequence obtained by 5’ RACE amplification. The control morpholino (MO) was 5’-CCTCTTACCTCAGTTACAATTTATA-3’. The MOs were designed and synthesized by Gene Tools Inc (Phiomath, OR, USA). Techniques for MO microinjection were as described previously [44]. For semiquantitative RT-PCR experiments, 0.5 mM *αA-crys* sbMO was injected into 1- to 4-cell-stage surface fish embryos and the embryos were cultured through 40 hr post-fertilization (hpf).

### *αA-crys* mRNA transcription

The full-length *aA-crys* coding region was amplified from surface fish cDNA using 5’-GGGGGATCCACACCTCCCTCCAGTTCTCTT-3’ (forward) and 5’-GGGTCTAGACCAGGTGAGGAAGACCTAGC-3’ (reverse) primers. The purified PCR product was digested with BamH1 and XbaI, ligated into the pCS2+ plasmid (a gift from Kandi Kero, NIH/NICHD), and the chimeric plasmid was confirmed by sequencing. After linearization with NotI, the capped mRNA was *in vitro* transcribed using the mMESSAGE mMACHINE Sp6 kit (Life Technologies). After transcription, the *αA-crys* mRNA was recovered by LiCl precipitation, washed with 70% ethanol, suspended in H_2_O, and stored at -20°C. The mRNA was microinjected into 1- to 2-cell-stage surface fish embryos in sterile water containing 0.05% phenol red.

### Terminal deoxynucleotidyl transferase dUTP nick end labeling (TUNEL)-mediated detection of apoptosis

MO-injected embryos and uninjected controls were assayed for apoptosis by TUNEL using the *In Situ* Cell Death Detection Kit (Roche, Mannheim, Germany) for whole-mount embryos. The embryos were fixed with 4% PFA and treated with Proteinase K (10 μg/ml) for 5 minutes prior to the assay. Then the fluorescent signal was transformed to a light signal with DAB Substrate (Roche), and the embryos were cleared in PBS/glycerol (1:1) for viewing with a light microscope.

## Results

### *αA-crys* gene knockdown promotes lens apoptosis

To investigate the relationship between *αA-crys* expression and lens apoptosis, we knocked down *αA-crys* expression by injecting surface fish embryos with either an *αA-crys* sbMO or a cocktail of tbMO and sbMO and determined the effects on lens and eye development at 40 hpf (Figure 1). The following controls were also done: first, the extent of the *αA-crys* knockdown was determined by semiquantitative RT-PCR; second, the expression of *mip* mRNA, which is not downregulated in the cavefish lens [17], was determined; third, the effects of injection with an MO unrelated to *αA-crys* (control MO) were determined; and finally the effects of co-injection with both the sbMO and *αA-crys* mRNA were determined. The semiquantitative RT-PCR results indicated that *αA-crys* mRNA levels were substantially reduced by *αA-crys* sbMO (Figure 1K). In contrast, *in situ* hybridization showed that *mip* mRNA expression in the lens was not affected in 47 of 50 embryos injected with *αA-crys* sbMO (Figure 1L, M), which is consistent with the specificity of the *αA-crys* knockdown. Surface fish embryos injected with control MO (n = 68) showed no effects on lens development (Figure 1A) or apoptosis (Figure 1G) and no embryos developed without a lens. However, 37% of 441 embryos injected with *αA-crys* tbMO and sbMO developed eyes with no detectable lens (Figure 1D), 6% developed eyes with a small lens (Figure 1C), and the remaining 57% developed eyes with a normal sized lens (Figure 1B). In some of these embryos, the ventral portion of the optic cup and retina was reduced in size (Figure 1C, H). Apoptotic cells were observed in the lens and in parts of the retina in the *αA-crys* MO-injected embryos (Figure 1I, N) but only background levels were present in the uninjected and the control MO-injected embryos (Figure 1G, H, N), despite the fact that TUNEL was capable of detecting DNA breakdown throughout the embryo after DNase treatment (Figure 1F). Co-injection of the *αA-crys* sbMO and *αA-crys* mRNA restored a normal sized lens (49% of 147 injections) and reduced the number of lens apoptotic cells to background levels (Figure 1E, J, N). The results show that *αA-crys* knockdown induces lens apoptosis and in some cases a smaller or absent lens in surface fish, suggesting a link between downregulated expression of this gene and eye degeneration.

### Sequence changes in the surface fish and cavefish *αA-crys gene loci*

**Figure 2:**
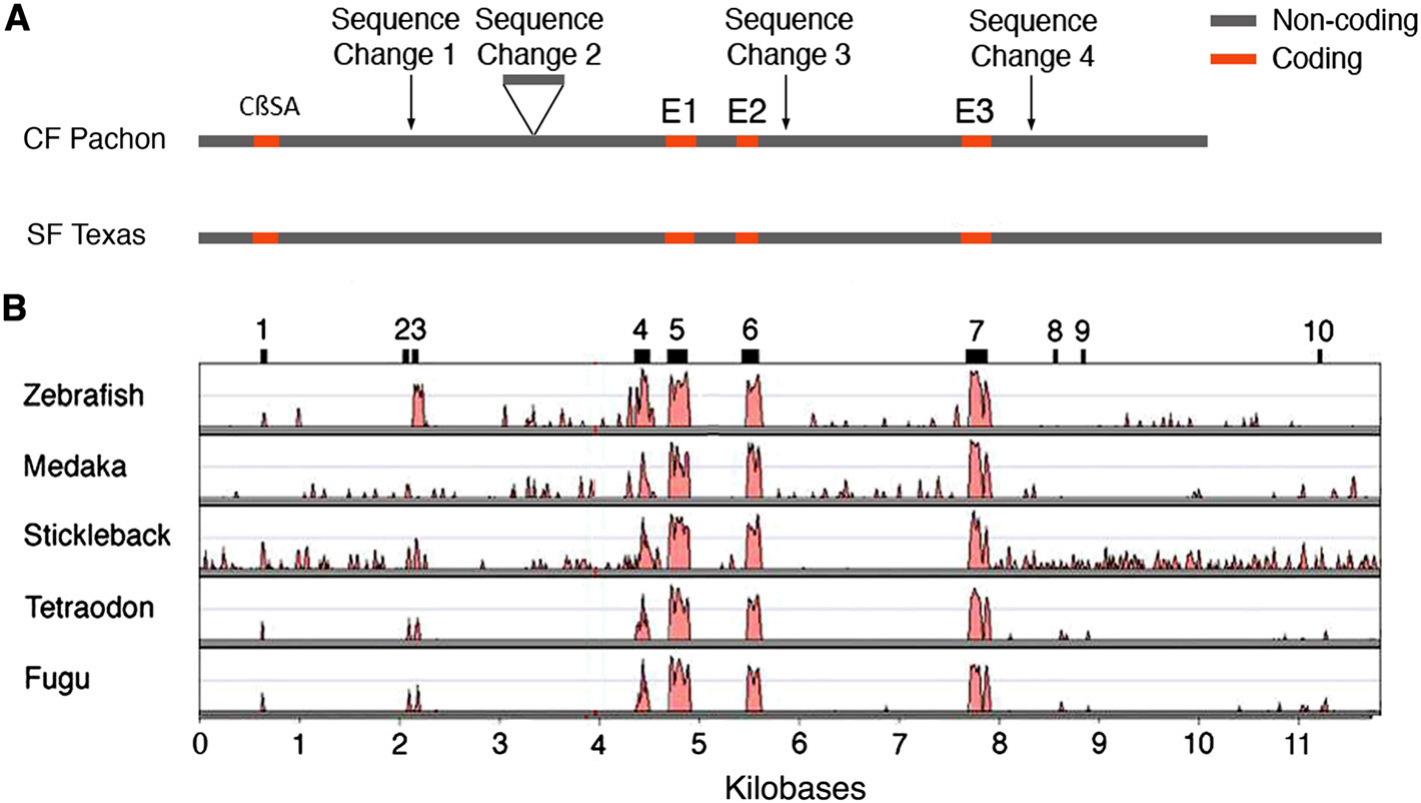
**Sequence changes in the surface fish and cavefish *αA-crys *gene loci. A**. A schematic diagram of the sequenced Texas surface fish (SF) and Pachón cavefish (CF) *αA-crys* loci showing the positions of four sequence changes (see Table 1). CßSA: last *cystathionine-ß-synthase a* exon*.* E1-3: *αA-crys* exons. **B**. Alignment of *αA-crys* loci in 5 teleost species with *Astyanax* Texas surface fish *αA-crys* shows 10 conserved regions (black bars labeled 1 to 10 on top of frame) but their sequences are not changed between surface fish and cavefish (Table 2).

To investigate the mechanism underlying *aA-crys* downregulation in cavefish, we searched for sequence changes (SC) in genomic DNA in and around the surface fish (Texas) and cavefish (Pachón) *aA-crys* gene loci. Behrens *et al*. [16] found only a few differences between the *Astyanax* surface fish and cavefish (Piedras population) *aA-crys* genomic DNA sequences in a 4.3 kb region beginning upstream of the putative promoter and ending in the coding region before the 3’ UTR. We sequenced about 10 kb of genomic DNA beginning in the last exon of *cystathionine-ß-synthase a* (*cßsa*), the gene immediately upstream of *aA-crys*, including the *aA-crys* exons, introns, and 5’ and 3’ flanking regions (Figure 2A).

**Figure 3:**
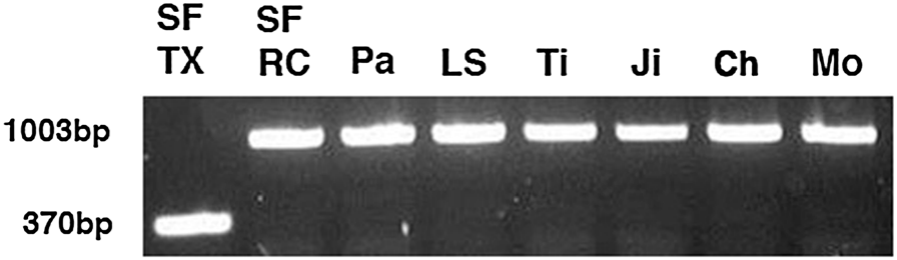
**PCR amplification of genomic DNA yields a 1,003-bp amplicon containing the 633-bp region (sequence change 2 in Figure**2**A) in Mexican (RC) surface fish (SF) and Pachón (Pa), Los Sabinos (LS), Tinaja (Ti), Jineo (Ji), Chica (Ch), and Molino (Mo) cavefish populations and a 370-bp amplicon lacking the 633-bp region in the Texas (Tx) surface fish population.** PCR amplification was carried out using primers flanking the 633-bp region in Pachón cavefish (see Methods).

The sequences of the Texas surface fish and Pachón cavefish *αA-crys* coding regions differed by a synonymous nucleotide substitution (G in surface fish and A in cavefish) located 18 nucleotides downstream from the translation start site, which was subsequently used as a specific marker for the Pachón cavefish *αA-crys* allele in hybrid embryos (see below). A few additional single base changes or gaps were scattered through the non-coding regions (data not shown). Four regions with more appreciable sequence differences were detected in the *aA-crys* non-coding regions (Figure 2A). These regions were investigated further by sequencing the corresponding PCR products from the Molino, Jineo, Chica, Los Sabinos, and Tinaja cavefish populations and a Mexican surface fish population (Table 1; Figure 3). The first change, which was located about 2.4 kb upstream of the *αA-crys* coding region (Figure 2A), was characterized by differences in the number of tandem CA repeats. The number of CA repeats in this region, which is likely to be a microsatellite locus, varied among all of the different surface fish and cavefish forms (Table 1). Thus, the first change is unlikely to represent a regulatory mutation in the cavefish *αA-crys* gene. The second change was an apparent 633-bp insertion in the Pachón cavefish genomic DNA that was absent in Texas surface fish (Figure 2A; Table 1). Although, the latter change seemed to be a promising candidate for a critical mutation because it was also present in all of the other cavefish populations, it was also subsequently identified in Mexican surface fish (Table 1; Figure 3) and thus is not specific to cavefish. The third and fourth SC were differences in the number of CT repeats in the second *αA-crys* intron, which were also reported by Behrens *et al*. [16] in the Piedras cavefish population, and a TAAAA insertion in the 3’ UTR of the Texas surface fish *αA-crys* gene respectively (Figure 2A; Table 1). The third difference was present in Mexican surface fish and to various extents in all of the cavefish populations but not in Texas surface fish, whereas the fourth difference was present in Texas surface fish but not in Mexican surface fish or in any other cavefish population besides Pachón (Table 1). Thus, the third and fourth SC are also unlikely candidates for regulatory sequences. Therefore, it is doubtful that any of the four major SC are responsible for *αA-crys* downregulation in cavefish and may instead be polymorphic variations among different *Astyanax* populations.

**Table 1:** A summary of the four sequence changes (SC) detected in different surface fish and cavefish populations

	**SC1**	**SC2**	**SC3**	**SC4**
SF Texas	C– – ––––––––CACACACACACACACACA - A - G	–	G–AAGCCCCCTTCCCCCCCTCCCCCCCC–TGCCT–C–T	ATAAAAA
SF Mexico	CCACACACACACACACACACACACACACA–A–G	+	G–AAGCCCCCCTT–––––––CCCCCCCC–TGCCTTC–T	A– – – – A
CF Pachón	C– – ––––––––CACACACACACA– – –––––A–G	+	G–AAGCCCCCCTCCCTCC––CCCCCCCC–TGCCT–C–T	A– – – – A
CF Molino	C– – ––––––––CACACACACACA– – –––––A–G	+	GGAAGCCCC–––CCCTCC––CCCCCCCCCTTGCTT––T	A– – – – A
CF Jineo	CCA– ––––––– CACACACACACACACACA - A - G	+	G–AAGCCCCCCTCCC–CC––CCCCCCT––TGC–TT––T	A– – – – A
CF Chica	ND	+	GGAAGCCCCCCT–––T–C––CCCCCCCC–TGCCTTCCT	A– – – – A
CF Los Sabinos	C– – –––––––– CACACACACACACACACA - A - G	+	GGAAGCCCCCCT–––TC–––CCCCCCCC–TGCCTTC–T	A– – – – A
CF Tinaja	C– – ––––––––– –CACACACACACACACACAAG	+	ND	A– – – – A

Dashes (-) indicate gaps. ND is not determined. For SC2 “+” is present and “–” is absent.

In another approach, we aligned the *αA-crys* genomic sequences of *Astyanax mexicanus* with multiple teleost species to identify highly conserved regions and searched for sequence differences between surface fish and cavefish in these conserved regions. For this comparison, a conserved element was defined as a stretch of more than 30 bp that shared 50% or more of its sequence with *Astyanax* surface fish (Texas) and at least one other teleost species. Ten sufficiently conserved regions were identified (Figure 2B), including the putative *αA-crys* promoter and the three *αA-crys* exons, but no differences in sequence were present between Texas surface fish and Pachón cavefish in those locations other than the substitution at coding position 18 described above (Table 2).

Although it is conceivable that critical differences in regulatory regions lie outside the sequenced regions, these results prompted us to consider alternative explanations for *αA-crys* downregulation in cavefish.

**Table 2:** **Sequence differences in conserved regions (identified in Figure**2**B) between Texas surface fish (SF) and Pachón cavefish (CF)**

	**Conserved sequence**	**Difference in SF and CF**
1	CCCTATAGATCATTTAAAAATCAATTGTTAATTATTGTACTTTTGTCTTTCTATCTTTCTCCCTCT	None
2	GCTGCCCTCTGTTTGCCGTGGCCCTGGGCTCTCTGGGCTGG	None
3	TAAGCCTCTCCAGCGGGCTGCGCAAAAGCCCACAGGGATTACCTTAATGCTGGCCTGCATGTGCCAGAGGGGAAGTCATCGGATGCAGGATCAGGG	None
4	GGTCTGGTTTGTTTTGGCAGAAGATCACTTTCCGGCGGTAGCAGCAGCTGGCAATCGTGCCACTGCCTGAAGCCGTTCAGGGCCAGACTGTCGTCCGCAGGTCAACTGTTCTGCTGACGCTGGTGTTCCCACCATGAGCTAATGCCATTCCAGAAAGATCCCCATGTAAGCCCCTCTTTCTGCCACACAGATATAAAGGCTGGAGGTGGCCAGGGCAGAAGGTAGCACACACCGTCTGCGGATCCCT	None
5	CAGTCATTATGGATATTGCCATCCAGCACCCCTGGTTCCGACGTGCCCTGGGCTATCCATCCCGCCTCTTTGACCAGTTCTTCGGGGAGGGCCTGTTTGACTATGACCTCTTCCCCTACGCCACCTCCACCGTCAGCCCTTATTATCGGTACTCGCTCTTCCGCAACTTCCTGGATTCCTCCAACTCGGGCATGTCTGAGGTAAACAG	Exon1 “AGC” to “AAC”
6	TTTCCTTTGGTTCTTCCCTAGGTGAGGTCTGACAGAGACAAGTTCATGGTCTACCTGGATGTGAAGCACTTCTCCCCCGAGGAACTCAATGTAAAGGTGGCAGAAGACTACGTGGAGATCCAAGGCAAGCATGGGGAGAGACAGGT	Exon2 None
7	AGGATGACCATGGCTACATCTCCCGCGAGTTCCACCGCCGCTACCGCCTGCCCTCCAACGTGGACCAGTCGGCCATCACCTGCACCCTGTCGGCCGATGGTCTGCTCACCATCTGTGGGCCCAAGTCAGGCGGCTCAGAGAGCGGCCGTGGAGACCGCAGCATTCCTGTCACCCGTGACGACAAGACTAACTCTACCCCCTcCTCTTAGGCCGCCTCAT	Exon3 None
8	CCTGTCCTGTAGCCTCTAGTTCTACTTATA	None
9	CACCTTTGCTAGTTTGCTAAAAAGAAATTAA	None
10	CCATCTCAACTCAAGGCTGAAGTCAAACCT	None

### *αA-crys* gene expression in hybrid embryos

**Figure 4:**
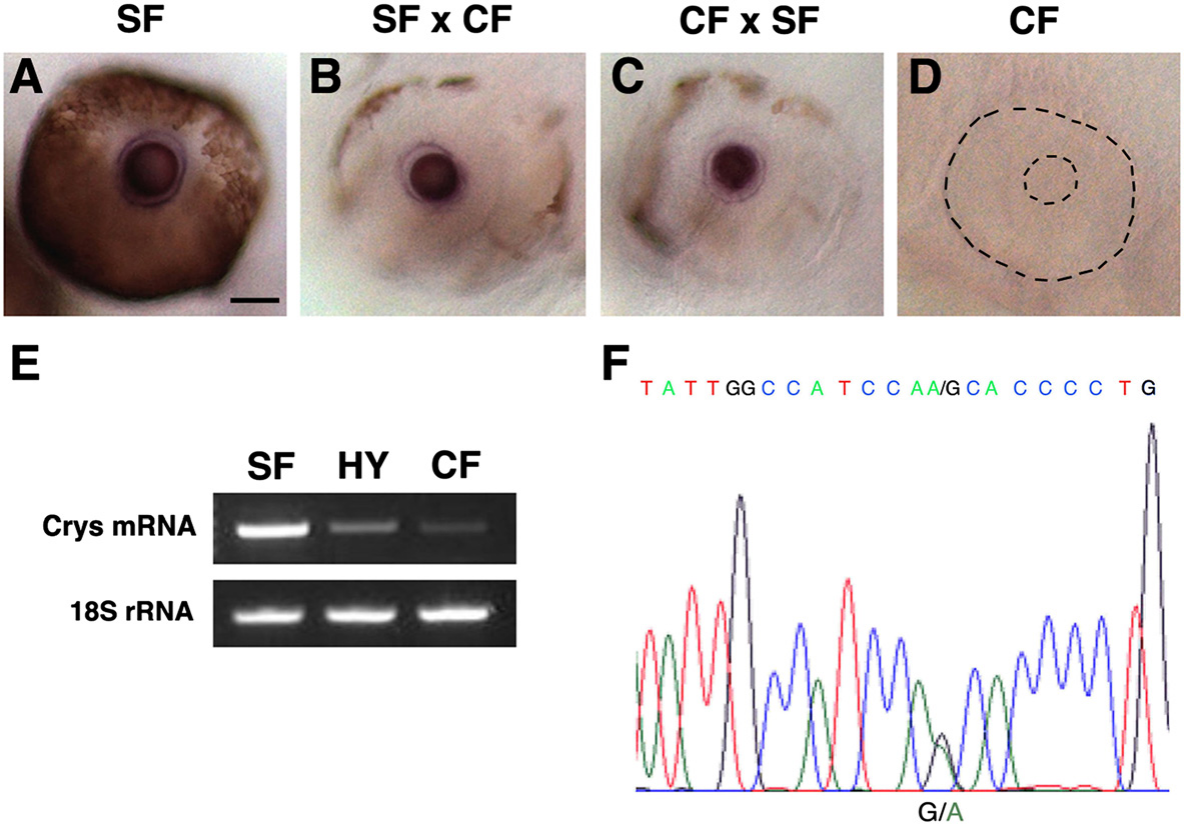
***αA-crys *expression is controlled by trans-acting factors in F1 hybrid embryos. (A-D)***In situ* hybridization showing *αA-crys* expression in the lens of **(A)** surface fish (SF), **(B)** surface fish X cavefish (CF) F1 hybrids, and **(C)** cavefish X surface fish F1 hybrids, but not in **(D)** cavefish embryos at 72 hr post fertilization (hpf). The eye and lens are outlined by dashes in cavefish **(D)**. Scale bar in A is 150 μm; magnifications are the same in **A**-**D**. **(E)** Semiquantitative RT-PCR showing *αA-crys* transcript levels in 40 hpf surface fish (SF), surface fish x cavefish F1 hybrid embryos (HY), and cavefish (CF) embryos compared to an 18S rRNA standard. **(F)** Profile of sequenced RT-PCR products from surface fish x cavefish F1 hybrid embryos showing a mixture of A (green) and G (black) residues at the first exon site distinguishing the surface fish from cavefish *αA-crys* alleles.

Trans-acting regulation is an alternative explanation for *αA-crys* downregulation in the cavefish lens. The consequences for cis- and trans-acting gene regulation would be different in F1 hybrid embryos [46]. For trans-acting regulation, hybrids would be expected to express both the surface fish and cavefish *αA-crys* alleles, whereas for cis-acting regulation the active surface fish *αA-crys* allele but not the inactive cavefish allele would be expressed. Therefore, we compared *αA-crys* expression in surface fish, cavefish, and their F1 hybrid embryos, which were produced by crosses in both directions (Figure 4). *In situ* hybridization detected *αA-crys* expression in surface fish but not in cavefish embryos (Figure 4A, D), confirming previous reports [16,17], and also in the lens of both surface fish (female) x cavefish (male) (Figure 4B) and cavefish (female) X surface fish (male) (Figure 4C) F1 hybrids. Semiquantitative RT-PCR showed higher *aA-crys* mRNA levels in hybrids compared to cavefish embryos, although the signal was below the level observed in surface fish embryos (Figure 4E).

The RT-PCR products were then sequenced to determine whether transcripts from both *αA-crys* alleles could be detected in hybrid embryos. As previously described, the *αA-crys* coding sequences of surface fish and cavefish are distinguished by a single nucleotide substitution, G in surface fish and A in cavefish (see above). Sequencing indicated either G or A at the critical site (Figure 4F), suggesting that both surface fish and cavefish *αA-crys* alleles are expressed in hybrid embryos. To substantiate these results, the RT-PCR products were cloned and sequenced. A total of 16 clones were selected randomly, and sequencing showed that 9 had A and 7 G at the position distinguishing the cavefish and surface *αA-crys* mRNAs (Table 3). These results suggest that the surface fish and cavefish *αA-crys* alleles are about equally active in hybrid embryos.

**Table 3:** **Aligned cloned sequences showing differences in position 18 (G or A) distinguishing the surface fish or cavefish *aA-crys *** **alleles in F1 hybrids**

**Number**	**F1 Hybrid**
1	TCCA**G**CACC
2	TCCA**G**CACC
3	TCCA**G**CACC
4	TCCA**G**CACC
5	TCCA**A**CACC
6	TCCA**A**CACC
7	TCCA**G**CACC
8	TCCA**A**CACC
9	TCCA**G**CACC
10	TCCA**A**CACC
11	TCCA**A**CACC
12	TCCA**A**CACC
13	TCCA**A**CACC
14	TCCN**A**CACC
15	TCCA**G**CACC
16	TCCA**A**CACC

Together with the genomic DNA sequencing results described above, the expression of the cavefish *αA-crys* allele in hybrids shows that cavefish contain a potentially functional *αA-crys* gene and suggests that trans-acting changes are involved in *aA-crys* downregulation.

### *sox2* gene expression during cavefish eye development

The expression of *crystallin* genes is regulated by the combinatorial activities of several different transcription factors, including Pax6 and Sox2 [40-42,47-49]. The *pax6* gene was previously shown to be downregulated in the cavefish lens [7,32], and this result was confirmed here (data not shown). Therefore, we focused on the *sox2* gene.

The full-length surface fish and cavefish *sox2* cDNA sequences were obtained by RACE extension of an initial *sox2* gene fragment isolated from surface fish RNA by RT-PCR. Alignment of the surface fish and cavefish *sox2* cDNA coding regions showed no nucleotide differences. Sox2 is a member of the Sox B1 family of transcription factors, which also includes the *sox1* and *sox3* genes [50,51]. All three Sox B1 family genes have regions of conserved sequence. Therefore, to obtain maximal *sox2* mRNA detection, probes for *in situ* hybridization were designed from two regions (*sox2*-A and *sox2*-B) within the single *sox2* exon [52,53] that showed minimal sequence homology with other Sox1B family members. Both *sox2*-A and *sox2*-B probes gave the same results in the experiments described below, and thus only the results obtained with *sox2*-A are shown.

**Figure 5:**
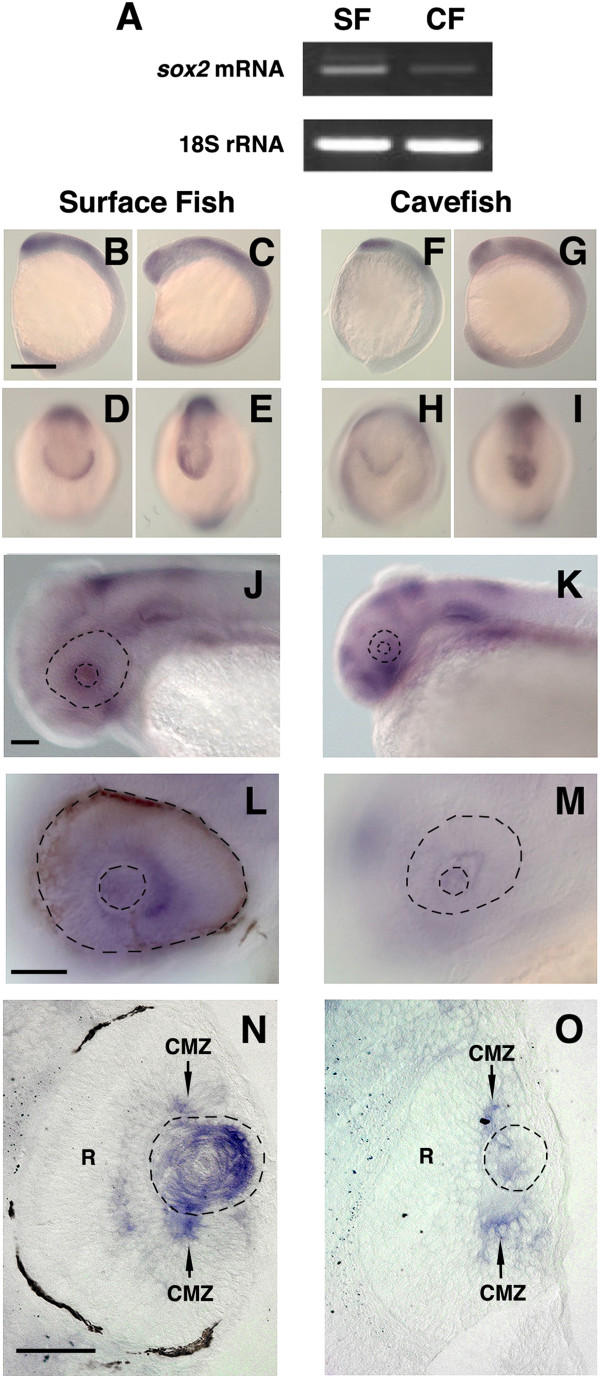
***sox2 *expression is downregulated in the lens during cavefish development. (A)** Semiquantitative RT-PCR showing reduced *sox2* mRNA levels in cavefish relative to surface fish embryos. **(B-O)***In situ* hybridization shows *sox2* expression during surface fish **(B-E, J, L, N)** and cavefish **(F-I, K, M, O)** development. **B**-**I** Tailbud **(B, D, F, H)** and 5-somite **(C, E, G, I)** stages viewed from the lateral **(B, C, F, G)** or rostral **(D, E, H, I)** sides. Scale bar in B is 250 μm; magnification is the same in **B**-**I**. **(J-O)***sox2* downregulation in the cavefish lens at 42 hr post fertilization (hpf). N and O are sections through the eye region of the embryos shown in **J**-**M**. Dashed lines indicate the eye and lens. R: retina. CMZ: ciliary marginal zone. Scale bar in **J**, **L** and **N** is 100 μm; magnification is the same in **J**, **K**; **L**, **M**; and **N**, **O**.

*In situ* hybridization revealed similar *sox2* expression patterns from the tailbud to the 5-somite stages with strong signal observed in the presumptive forebrain, hindbrain, spinal cord, and developing optic vesicle (Figure 5B-I). This is similar to the *sox2* expression patterns reported in other vertebrates [34,35,54]. Semiquantitative RT-PCR indicated that *sox2* mRNA levels were slightly reduced in cavefish embryos at 40 hpf (Figure 5A), and *in situ* hybridization showed that *sox2* expression was much weaker in the cavefish lens, although it was similar in the brain and otic regions in both *Astyanax* morphs (Figure 5J-M). Sections of the *in situ* hybridized embryos confirmed *sox2* downregulation in the cavefish lens. In contrast, *sox2* mRNA levels were about the same in the ciliary marginal zones (CMZ) of the retina in both morphs (Figure 5N, O). We therefore conclude that *sox2* expression is downregulated in the cavefish lens.

### *sox2* gene knockdown abolishes *αA-crys* expression and promotes lens apoptosis

**Figure 6:**
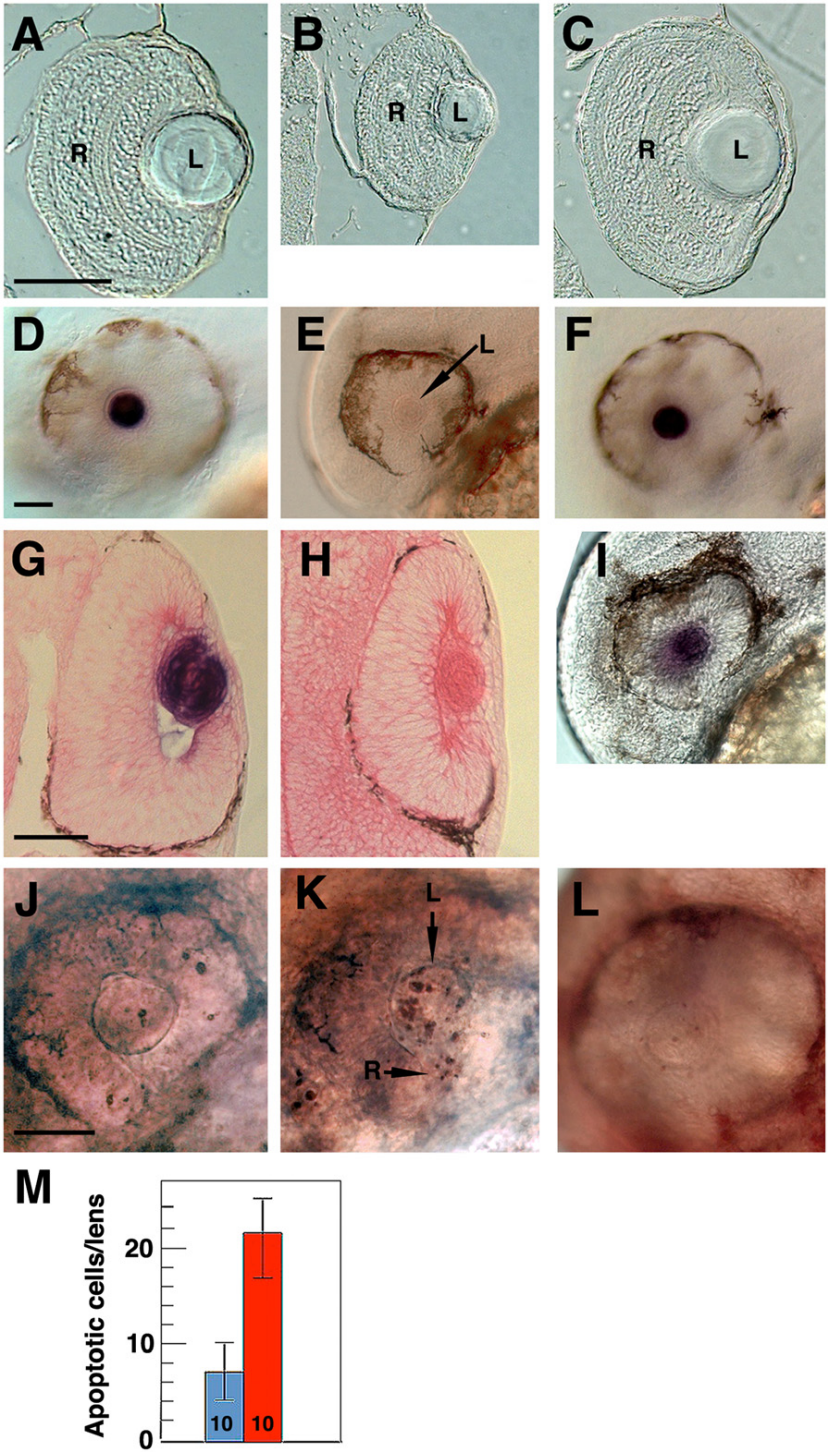
***sox2 *knockdown. (A-C)** Effects of *sox2* MO on eye development at 42 hr post fertilization (hpf). Sections of uninjected **(A)**, *sox2* MO injected **(B)**, and control MO-injected **(C)** embryos show reduced eye size after *sox2* knockdown. Scale bar in **A** is 150 μm: **A**-**C** are the same magnifications. **(D-H)***sox2* knockdown abolishes lens *αA-crys* gene expression. *In situ* hybridization of uninjected **(D)**, *sox2* MO-injected **(E)**, and control MO-injected **(F)** embryos shows no lens *αA-crys* expression after *sox2* knockdown. Sections through the eyes of *in situ* hybridized uninjected **(G)** and *sox2* injected **(G)** embryos confirm the absence of *αA-crys* expression in the *sox2* morphant lens. R: retina. L: lens. **(I)***In situ* hybridization showing lens *mip* gene expression in *sox2* MO-injected embryos. **(J-L)** Effects of *sox2* gene knockdown on apoptosis at 42 hpf. Terminal deoxynucleotidyl transferase dUTP nick end labeling (TUNEL) assays of un-injected **(J)**, *sox2* MO-injected **(K)**, and control MO injected **(L)** embryos show apoptosis in the lens (arrow labeled L) and retina (arrow labeled R) in *sox2* morphants. Embryos are shown at 42 **(A-I)** and 48 **(J-L)** hpf. Scale bars in **D**, **G**, and **J** are 100 μm; magnification is the same in **A-C**; **D-F**, **I**; **G** and **H**; and **J**-**L**. **M**. Histogram showing apoptotic cells in the lens of uninjected embryos (blue) and *sox2* MO-injected embryos (red). Error bars represent SD. Numbers at the base of the histogram represent sample sizes. *P* = 0.00, one-way analysis of variance (ANOVA).

To determine the effects of *sox2* on *αA-crys* expression and lens apoptosis, we knocked down the single exon *sox2* gene by injecting surface fish embryos with a tbMO (*sox2* MO) and determined the effects on *αA-crys* expression and apoptosis at 40 hpf. Some embryos were injected with control MO, and the effects of *sox2* MO injection were determined on *mip* gene expression as well. The *sox2* MO-injected embryos showed smaller eyes than controls (Figure 6A-C). Although *sox2* morphants developed a normal appearing eye and lens (Figure 6B, E, H), *αA-crys* expression was lost in 85 of 89 *sox2* morphant lenses (Figure 6E). In contrast, strong *αA-crys* expression was detected in the lens of uninjected and control morphant embryos (Figure 6D, F). Furthermore, *mip* gene expression was unaffected in *sox2* morphant embryos (n = 123; Figure 6I; also see Figure 1L, M). TUNEL analysis showed that the injection of *sox2* MO but not the control MO induced apoptosis in the surface fish lens (Figure 6J-L). Apoptotic cells were also detected in parts of the retina (Figure 6K) and brain (data not shown). The results show that *sox2* knockdown abolishes *αA-crys* expression and induces apoptosis in the surface fish lens.

## Discussion

During cavefish evolution, eyes were lost through lens apoptosis, which also promotes degeneration in some of the surrounding optic tissues [8]. *αA-crys*, which is strongly downregulated in the cavefish lens [16,17] and located in one of the genomic regions (QTL) responsible for eye loss [13], has been proposed as a candidate for regulating the evolution of cavefish eye degeneration. The purpose of this study was to determine the role of the *αA-crys* gene in cavefish eye regression and to understand how this evolutionary change in gene activity is controlled.

### Role of *αA-crys* in lens apoptosis and eye degeneration

Previous studies of eye degeneration in *Astyanax* used lens transplantation to show that apoptosis is controlled autonomously within the lens vesicle [8]. When a surface fish embryonic lens was transplanted into a cavefish optic cup, apoptosis and eye degeneration was prevented. Conversely, when a cavefish embryonic lens was transplanted into a surface fish optic cup apoptosis occurred on schedule and eye growth was reduced. As the surface fish lens can stimulate cavefish eye growth and differentiation, and cavefish have retained the ability to respond to a lens inductive signal, then some of the factors responsible for eye degeneration must be intrinsic to the cavefish lens*.* Moreover, the discovery of multiple QTL responsible for the *Astyanax* lens phenotype [1,11-13] suggests that several different factors that function individually or redundantly are involved in lens apoptosis. Our results suggest that *αA-crys* is one of these factors.

MO-based knockdown of the *αA-crys* gene in surface fish showed that apoptosis was induced in the lens and that this effect could be rescued by co-injection with *αA-crys* mRNA. Apoptosis also occurred in parts of the retina after *αA-crys* knockdown, probably as a secondary effect of lens dysfunction. During cavefish development apoptosis begins in the lens and is followed by the appearance of apoptotic zones in the retina [7,9,10]. In some of the *αA-crys* morphants, the size of the ventral portion of the retina was reduced, a phenotype that is also typical of the cavefish optic cup [8,55]. Thus, our results indicate that *αA-crys* knockdown in *Astyanax* surface fish induces lens and retina phenotypes similar to those that occur naturally in cavefish. This is further evidence that *αA-crys* downregulation is one of the causes of eye degeneration during *Astyanax* cavefish evolution.

Despite the induction of lens apoptosis, a normal sized lens developed in some embryos after *αA-crys* knockdown. This result may have been obtained because of incomplete knockdown of the *αA-crys* gene or because other survival factors with redundant functions are operating in surface fish. We favor the latter hypothesis because lens development appears to be completely arrested in cavefish even through there is residual *αA-crys* gene activity. It is possible that *αB-crys*, which together with *αA-cry* composes the α-crystallin chaperone and also has anti-apoptotic activity [20], may be one of these redundant factors. A similar explanation may apply to a recent report that MO-induced *αA-crys* knockdown has no effects on lens development in zebrafish [56].

### Molecular basis of *αA-crys* downregulation in cavefish

The association of the *αA-crys* gene locus with an eye QTL [13,16] has suggested the possibility that a cis-acting mutation in this gene could be important in cavefish eye regression. Although we show here that *αA-crys* is indeed involved in eye degeneration through its effects on lens apoptosis, no obvious cis-acting changes were revealed that are likely to be responsible for the downregulation of this gene. We found four sequence changes in the *αA-crys* non-coding regions of Pachón cavefish relative to Texas surface fish, but none of these were located in conserved regions with known cis-acting regulatory elements in teleost *αA-crys* genes*.* Three of these changes were polymorphic variations in repeated sequences that are unlikely to have regulatory effects on *αA-crys* expression, whereas an apparent 633 bp insertion in the *αA-crys* 5’ non-coding region of Pachón cavefish and many other cave populations was also found in eyed Mexican surface fish, conclusive evidence against its role in *αA-crys* regulation. The 633-bp change suggests that caution should be exercised in comparisons of sequence information between the Texas surface fish and other *Astyanax* populations.

Since enhancer elements can be located considerable distances from the coding region in vertebrate genes, we cannot exclude the possibility that cis-acting changes involved in transcriptional regulation are located outside of the sequenced 10-kb region. However, transcription of the cavefish *αA-crys* allele in F1 hybrids points to a functional gene with trans-acting control. We took advantage of a synonymous substitution in the first *αA-crys* exon to test the functionality of the cavefish *αA-crys* promoter in a hybrid background [46]. F1 hybrids inherit one copy of the *αA-crys* gene from each parent. Therefore, if transcripts of both cavefish and surface fish *αA-crys* are detected in F1 hybrids, then the *αA-crys* cis-acting regulatory information inherited from the cavefish parent must be functional in the surface fish trans-acting background. Roughly equal levels of *αA-crys* transcripts originating from the cavefish and surface fish alleles were detected in F1 hybrids, suggesting that cis-acting changes do not impede transcription of the cavefish *αA-crys* gene. Thus, we conclude that trans-acting rather than cis-acting regulation is the best explanation for *αA-crys* downregulation in cavefish.

### *αA-crys* as an eye QTL candidate gene

One of the reasons for focusing on the *αA-crys* gene was its association with a significant *Astyanax* eye QTL [13]. The fact that *αA-crys* is strongly downregulated in two different cavefish populations [16,17] provided further evidence for a direct role of this gene in the cavefish eye phenotype. However, *αA-crys* is unlikely to harbor a mutation responsible for cavefish eye degeneration. The eye QTL associated with the *αA-crys* locus is expected to contain many genes. Instead of *αA-crys* one or more of the other genes linked to this QTL may define its association with cavefish eye degeneration. The F1 hybrid test devised here for determining the relative roles of trans-and cis-acting factors in cavefish *αA-crys* gene regulation could be helpful in deciding whether other candidate genes within eye QTL have a cis-acting mutation.

### Role of *sox2* in lens apoptosis and eye degeneration

Our search for a trans-acting candidate gene involved in *αA-crys* regulation focused on *sox2*. The Sox2 transcription factor functions together with Pax6 to regulate *crystallin* gene expression during vertebrate eye development [40,41]. Previous studies showed that Pax6 is downregulated in the cavefish lens, particularly during the latter stages of eye development [32], but prior to this investigation the status of *sox2* in developing cavefish had not been determined. Similar *sox2* expression patterns were detected in early surface fish and cavefish embryos by *in situ* hybridization, with strong signals in the presumptive forebrain, hindbrain, spinal cord, and developing optic vesicle. By about a day after hatching, however, *sox2* expression was much weaker in the lens of cavefish relative to surface fish larvae, despite similar expression in the brain, retina, and the otic regions of both morphs. Specific *sox2* downregulation was confirmed in cavefish by semiquantitative RT-PCR. Thus, we conclude that the *sox2* gene is downregulated in the cavefish lens.

The downregulation of *sox2* could occur because of specific effects on this gene or because of apoptosis and the removal of the *sox2*-expressing cells themselves from the cavefish lens. We favor the former possibility because our results show that *sox2* knockdown induces lens apoptosis in surface fish. Contrary to the case of *αA-crys*, though as expected from broad *sox2* expression throughout the central nervous system, *sox2* knockdown also induced apoptosis in parts of the retina and brain. These regions are not affected by *sox2* downregulation in cavefish due to its restriction to the lens. Thus we propose that the effects of *sox2* on lens apoptosis are mediated through downstream control of *αA-crys* expression. This interpretation is strongly supported by our demonstration that *sox2* knockdown abolishes *αA-crys* expression in the surface fish lens.

A primary function of Sox2 is stem cell maintenance [57,58]. Therefore, *sox2* downregulation could explain previous findings concerning the relative contributions of cell proliferation and apoptosis in the cavefish lens [10]. PCNA labeling showed that cell division and apoptosis are not balanced in the cavefish lens; the latter eventually overwhelms the former, resulting in striking differences between lens and body growth during larval development. Lens stem cells generate new fiber cells from a proliferation zone in the lens epithelial layer. Accordingly, in concert with inducing lens apoptosis via *αA-crys*, *sox2* downregulation could also affect the generation of new fiber cells. The proposed effects of *sox2* on cell proliferation in the lens contrast sharply with its effects in the CMZ, where most new retinal cells are generated. BrdU and PCNA labeling of cavefish embryos have shown that cell division levels are not altered in the CMZ [9,59], although retinal growth is reduced because newly born progenitor cells undergo cell death rather than differentiation [10]. Thus, *sox2* expression in the cavefish CMZ is consistent with the continuation of stem cell maintenance and progenitor cell proliferation in the retina.

We propose that *sox2* downregulation in the cavefish lens has two consequences. First, stem cell maintenance and production of new lens fiber cells is curtailed. Second, lens apoptosis is induced via effects on *αA-crys* expression. Together, these two factors may be responsible for the arrest and eventual degeneration of the lens in blind cavefish.

### The *sox2*-*αA-crys* pathway for lens survival

The results suggest that a genetic pathway leading from *sox2* to *αA-crys* is required for lens survival in *Astyanax* surface fish, and that defects in this pathway trigger lens apoptosis and eye degeneration. Two lines of evidence support this pathway: (1) *αA-crys* expression is abolished by *sox2* knockdown, indicating the *sox2* gene functions upstream of *αA-crys* in the lens, and (2) lens apoptosis can be induced either by *sox2* or *αA-crys* knockdown, indicating that both genes are required for normal lens development. This pathway is also consistent with results showing that αA-crys promotes a healthy lens by suppressing the apoptotic proteins caspase-3 and Bax during mouse development [21]. We are uncertain whether *sox2* controls *αA-crys* directly or indirectly via downstream effects on one or more of the other transcription factors regulating the *αA-crys* gene. We also do not know where the defect in the cavefish pathway is located. Mutations could occur in a cis-acting region of the *sox2* gene controlling *αA-crys* expression or in a trans-acting gene functioning upstream of *sox2*. Examination of the assembled cavefish genome [McGaugh and 20 others 2014, The cavefish genome reveals candidate genes for eye loss, In submission] does not reveal an eye QTL linked to the *sox2* gene. Therefore, it is possible that mutation in a trans-acting gene (or genes) controlling *sox2* expression is responsible for interrupting this pathway. The cavefish and surface *sox2* alleles identified in the present investigation did not show any sequence changes, preventing the use of the F1 hybrid method we developed for testing *αA-crys* to resolve this issue.

## Conclusions

We conclude that a lens survival pathway including the *sox2* and *αA-crys* genes is required for normal lens and eye development in *Astyanax* surface fish. Gene knockdown analysis indicates that *sox2* functions upstream of *αA-crys* in this pathway. Downregulation of either gene results in lens apoptosis and abnormal development of lens-dependent optic tissues. Hybrid analysis shows *αA-crys* downregulation in cavefish is caused by an evolutionary change in an upstream gene in the lens survival pathway, either *sox2* itself or a gene regulating *sox2*. An unknown upstream mutation in the lens survival pathway may be one of the factors responsible for the evolution of eye degeneration in blind cavefish.

## Abbreviations

Bp: base pair(s); CMZ: ciliary marginal zones; hpf: hour post-fertilization; MABT: 150 mM maleic acid 100 mM NaCl, pH7.5, 0.1% Tween-20; MO: morpholino; PFA: paraformaldehyde; PBS: phosphate-buffered saline; PBST: phosphate-buffered saline plus 0.1% Tween; PTU: phenylthiourea; QTL: quantitative trait loci; SC: sequence change; sbMO: splice-blocking morpholino; SSCT: saline sodium citrate plus 0.1% Tween-20; tbMO: translation-blocking morpholino; TUNEL: terminal deoxynucleotidyl transferase dUTP nick end labeling; UTR: untranslated region.

## Competing interests

The authors declare that they have no competing interests.

## Authors’ contributions

WRJ and LM conceived the study and all authors participated in designing the project. LM performed all of the experimental studies. WRJ performed the statistical analyses. All authors wrote, read, and approved the final manuscript.
